# Advancements in Cortisol Detection: From Conventional Methods to Next-Generation Technologies for Enhanced Hormone Monitoring

**DOI:** 10.1021/acssensors.3c01912

**Published:** 2024-03-29

**Authors:** Visesh Vignesh, Bernardo Castro-Dominguez, Tony D. James, Julie M. Gamble-Turner, Stafford Lightman, Nuno M. Reis

**Affiliations:** † Department of Chemical Engineering and Centre for Bioengineering and Biomedical Technologies (CBio) University of Bath, BA2 7AY Bath, U.K.; ‡ Department of Chemical and Engineering and Digital Manufacturing and Design University of Bath, BA2 7AY Bath, U.K.; § Department of Chemistry, University of Bath, BA2 7AY Bath, U.K.; ∥ Department of Psychology, Bournemouth University, BH12 5BB Bournemouth, U.K.; ⊥ Translational Health Sciences, Bristol Medical School, University of Bristol, BS1 3NY Bristol, U.K.

**Keywords:** cortisol, stress, immunoassay, continuous, biorhythm, electrochemistry, point-of-care, rapid

## Abstract

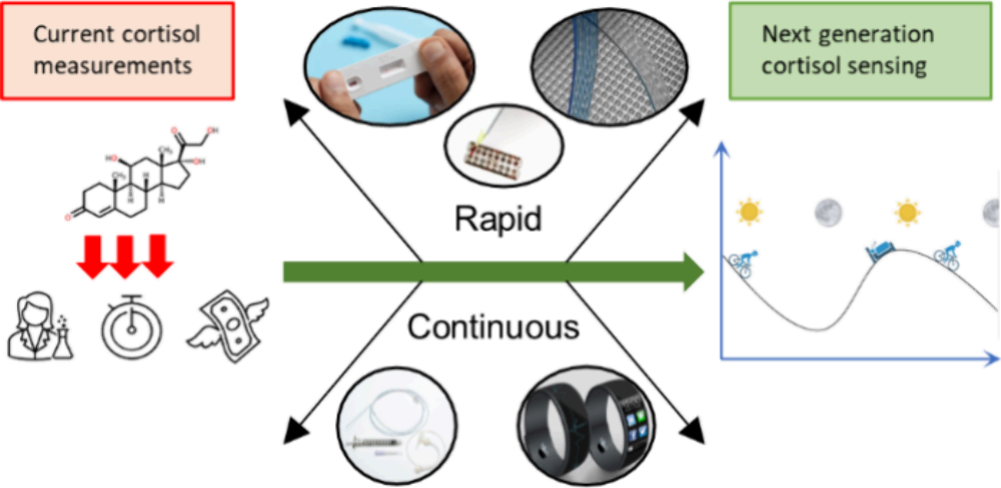

The hormone cortisol, released as the end-product of the hypothalamic-pituitary-adrenal (HPA) axis, has a well-characterized circadian rhythm that enables an allostatic response to external stressors. When the pattern of secretion is disrupted, cortisol levels are chronically elevated, contributing to diseases such as heart attacks, strokes, mental health disorders, and diabetes. The diagnosis of chronic stress and stress related disorders depends upon accurate measurement of cortisol levels; currently, it is quantified using mass spectroscopy or immunoassay, in specialized laboratories with trained personnel. However, these methods are time-consuming, expensive and are unable to capture the dynamic biorhythm of the hormone. This critical review traces the path of cortisol detection from traditional laboratory-based methods to decentralised cortisol monitoring biosensors. A complete picture of cortisol biology and pathophysiology is provided, and the importance of precision medicine style monitoring of cortisol is highlighted. Antibody-based immunoassays still dominate the pipeline of development of point-of-care biosensors; new capture molecules such as aptamers and molecularly imprinted polymers (MIPs) combined with technologies such as microfluidics, wearable electronics, and quantum dots offer improvements to limit of detection (LoD), specificity, and a shift toward rapid or continuous measurements. While a variety of different sensors and devices have been proposed, there still exists a need to produce quantitative tests for cortisol — using either rapid or continuous monitoring devices that can enable a personalized medicine approach to stress management. This can be addressed by synergistic combinations of technologies that can leverage low sample volumes, relevant limit of detection and rapid testing time, to better account for cortisol’s shifting biorhythm. Trends in cortisol diagnostics toward rapid and continuous monitoring of hormones are highlighted, along with insights into choice of sample matrix.

Cortisol is the primary glucocorticoid hormone released by the body in response to stress,^1,2^ with chronic cortisol levels leading to various pathophysiologies.

This hormone is regulated by the hypothalamic–pituitary–adrenal (HPA) axis, a complex neuroendocrinological system (Figure 1A). Most cortisol in blood (90%) is bound to a carrier protein, cortisol binding globulin (CBG), with free cortisol only making up 5–10% of the total cortisol in circulation.^3^ Only free cortisol is biologically active, with bound cortisol being physiologically inactive. Cortisol controls a wide range of physiological processes, such as promoting gluconeogenesis, reducing inflammation, suppressing the immune system^4^ and modulating cognitive processes.^5^ These responses anticipate and assist the body’s adaptation to stressful conditions by providing energy for awakening, fuelling a “fight or flight” response, and diverting resources to deal with a stressor.^2^

**Figure 1:**
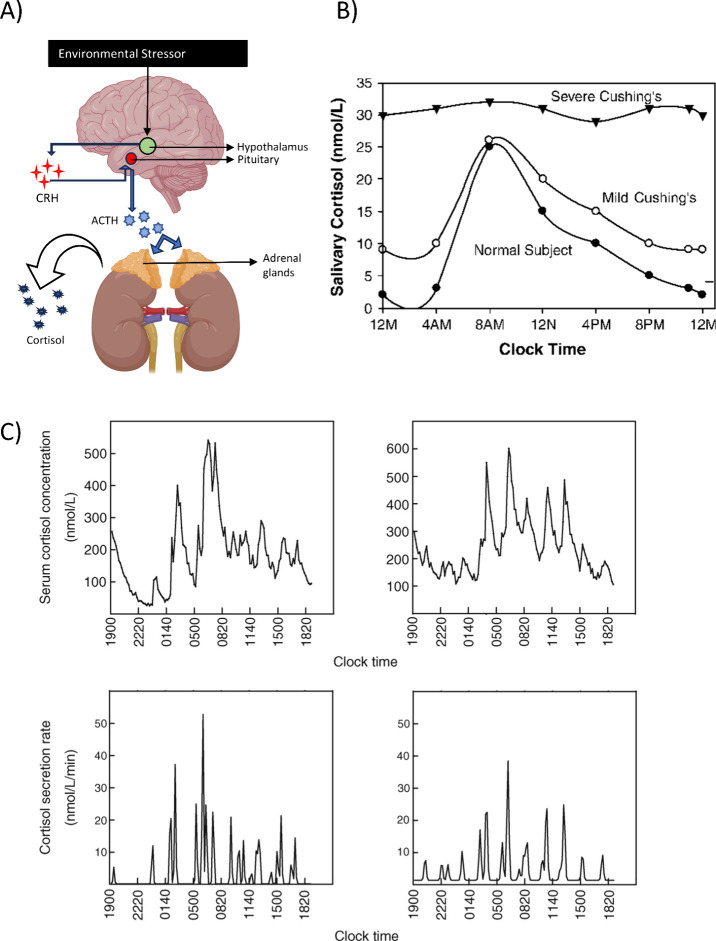
(A) Overview of the HPA axis. Created with Biorender. (B) Graph comparing normal cortisol levels versus mild and severe Cushing’s syndrome. Reproduced with permission from ref (113). Copyright 2005, Endocrinology and Metabolism Clinics of North America. (C) Rate of cortisol secretion versus clock time, depicting cortisol rhythm being made up of pulsatile ultradian secretions. Reproduced with permission from ref (114). Copyright 2009, *Journal of Medical Engineering and Technology*.

Upon detecting a stressor through the central nervous system (CNS), cells situated in the hypothalamus are stimulated to secrete corticotrophin-releasing hormone (CRH). CRH then stimulates the release of adrenocorticotrophin-releasing hormone (ACTH), which is recognized by cells in the adrenal cortex, to produce cortisol.^6^ The cascade is regulated by a negative feedback loop,^7^ with high cortisol levels signaling to the hypothalamus to halt further CRH secretion, as well as to the pituitary gland to halt ACTH production. Cortisol follows a well-characterized diurnal rhythm,^8^ with a peak level observed shortly after awakening, dipping to its lowest point at midnight before starting to rise again during the nocturnal hours. This rhythm is composed of multiple ultradian cycles,^9^ each cycle typically lasts 60–120 min and is independent of external stressors, with additional cortisol secreted in response to stressors. The rhythmicity of cortisol secretion is one of its defining traits, with research showing detectable cortisol rhythms in infants as young as one month of age.^10^

When cortisol production becomes overactivated, baseline cortisol levels in the body can become dysregulated, leading to impairment of normal body functioning and triggering diseases such as diabetes mellitus and depression.^11^ Excessive cortisol levels in blood are a diagnostic factor for Cushing’s syndrome, where patients may exhibit hyperglycaemia, muscle atrophy, delayed wound healing and increased susceptibility to infections.^12^ Conversely, lowered cortisol levels can lead to a state of adrenal insufficiency, also called Addison’s disease. In this condition, cortisol levels are lowered, leading to a decrease in glucocorticoid hormone levels and a corresponding upsurge in adrenocorticotropic hormones. The disease can be life threatening if left unchecked, leading to hypotension and volume depletion or adrenal crisis.^13^ The symptoms of these disorders overlap strongly with other diseases,^14^ making the assessment of an individual’s baseline cortisol levels key to diagnosing these conditions in a timely manner. Another factor that can influence cortisol measurement is the age of the person being sampled. Compared to adults, children show a much greater variability in cortisol rhythms through the day.^15^ The exact reason for this is unknown, as the HPA axis reaches adult-level maturity within the first four years of development. Nevertheless, it is important to consider differential cortisol expression in adults versus children when conducting population wide studies.

In addition to physiological insult, chronic psychosocial stress has been associated with dysregulation of cortisol levels and linked to poorer physical as well as mental health outcomes. In a study on relatives of patients in an intensive care unit (ICU), higher cortisol levels were linked to avoidance behavior and a depressed state.^16^ While results from the literature are confusing, cortisol levels seem to be elevated in some patients suffering from depression.^17^ Previous research has also suggested dysregulated cortisol profiles in children with autism, revealing elevated levels in the evening and lowered cortisol in the morning.^18,19^

Given the negative effects of chronic stress, there is a need to measure cortisol levels accurately and reliably. As early as 1954, studies have revealed the detrimental effects of raised cortisol levels in mice and patients undergoing corticosteroid therapy.^20−22^ The effects of cortisol on the human body were particularly scrutinized in the case of elite athletes, where minor differences in hormonal makeup can mean the difference between victory and defeat.^23^ More recently, a growing understanding of stress and the role of cortisol in pivotal processes such as catabolism and sleep hygiene show that there is a need to measure cortisol levels accurately and reliably, with tangible benefits for a general population.^24,25^

Nowadays, the most common method of measuring cortisol levels is via immunoassay,^27^ with a wide range of kits available commercially for centralized diagnostic purposes. The current gold standard for cortisol measurement is mass spectroscopy (MS), a highly sensitive and precise technique for small molecule detection, which also has the advantage of being able to measure multiple compounds from the same sample.^28^ While both immunoassays and MS offer unique advantages and disadvantages, they are both difficult to adopt to point-of-care (PoC) detection, due to factors such as cost, requiring specialized personnel and overhead time loss. Additionally, neither of these modalities can be used for continuous monitoring of cortisol.

As interest in cortisol biosensing has steadily increased in recent years, there is now a vast amount of information regarding various aspects of cortisol sensing. This Review summarizes and critically reviews the most remarkable advances in the last 5 years in the area of cortisol sensing and highlights key technologies that could facilitate a shift from current lab-based cortisol measuring to rapid and/or continuous sensing at the point-of-need or point-of-care. Several important reviews have previously discussed certain specific aspects of cortisol measurements such as immunoassays^29^ or wearables.^30^ Herein, we present a broader overview of cortisol sensing, from biology and pathophysiology to continuous monitoring and cutting-edge biosensing technologies, helped by the interdisciplinary expertise of our team, including a health psychologist, endocrinologist, molecular chemist and microfluidic and biosensing experts. We provide the reader with a background on cortisol biology and the difficulties associated with its detection, followed by a reflection on advantages and challenges for utilizing various sample matrices. We then followed with a critical overview of established techniques for cortisol sensing, such as microdialysis, that were discussed along with their shortcomings for clinical use. We then finished with a critical assessment of the transformative nature of switching from current lab-based measurement of cortisol to a rapid and/or real-time cortisol sensing.

## Cortisol Sampling

The first aspect that must be considered in terms of cortisol measurement is sampling and the challenges presented in different matrices. It is well established in literature that cortisol is present in all major bodily fluids, including sweat, saliva, interstitial fluid, and blood (serum). This plethora of potential samples offers a great deal of flexibility when designing a detection modality for cortisol, with each offering differing advantages and disadvantages, summarized below in Table 1 and herein discussed.

**Table 1:** Comparison of Various Samples for Cortisol Detection with Their Respective Advantages and Disadvantages^4,108,109^

Sample fluid	Invasive sampling	Correlation with circulating levels	Free cortisol	Ease of collection	Preprocessing required	Can be collected during sleep	Typical cortisol range
Saliva		√	√	√	√		5.27 ng/mL^110^
Sweat		√		√			8–140 ng/mL^65^
Serum	√				√		50–163ng/mL^111^
Interstitial fluid	√	√	√			√	1–11 ng/mL^112^

### Serum

Serum cortisol sampling gives a measure of total (free plus bound) cortisol,^31^ and is used to determine the total serum cortisol level of a patient. Serum cortisol can more accurately reflect rapid cortisol changes dynamically, as opposed to a matrix such as saliva which requires time for cortisol diffusion from blood.^32^ However, there are numerous reported challenges when attempting to use serum as the analyte fluid. Apart from the extraction process being time and labor intensive, prefiltration steps are often required, using techniques such as dialysis, ultrafiltration, and gel filtration to separate bound and unbound cortisol fractions. Additionally, the venepuncture procedure required to collect blood samples is an invasive process, which in some patients can trigger increased cortisol synthesis,^33^ potentially providing misleading readings.

### Saliva

Salivary cortisol offers the opportunity to measure free cortisol due to passive diffusion. The concentration of salivary cortisol is unaffected by flow rate from the salivary glands and offers the benefits of measuring biologically active cortisol without resorting to invasive serum sampling.^34^ Saliva is readily (and plentifully) available as a sample fluid and can be rapidly extracted with a swab, passive drool techniques, or cuvettes.^35^

Having said that, one of the biggest hurdles in measuring cortisol from saliva is the presence of salivary cortisone, the inactive form of cortisol. Cortisone cross reacts with cortisol specific binding agents in immunoassays and can create background noise. Furthermore, cortisol is rapidly converted into cortisone by the salivary glands due to the presence of an enzyme, 11-β-dehydrogenase isozyme 2, leading to reduced levels of cortisol in saliva than in serum. Oral hydrocortisone treatments contaminate the salivary cortisol pool, leading to uninterpretable readings for patients undergoing such treatment.^36^ Finally, patients should fast for 30 min prior to sample collection, making saliva an unwieldy choice for multiple/continuous cortisol sampling, or when a subject is asleep. The lower concentration of cortisol present in saliva, approximately 1 order of magnitude lower than that of other matrices (Table 1) is a major challenge when it comes to limit of detection (LoD) of the biosensor.

### Interstitial Fluid

Interstitial fluid is the liquid solution surrounding tissue cells, providing nutrients that passively diffuse into the cells, while simultaneously removing waste products of metabolism such as carbon dioxide. The fluid contains free cortisol in detectable concentrations, and is a better indicator of cortisol levels in tissue, as opposed to serum cortisol.^37^ One of the key advantages over other matrices such as saliva, is the possibility of sampling interstitial fluid in a continuous manner, via methods such as microdialysis,^38^ also reviewed in this article.

### Sweat

Like saliva, sweat is an easily accessible fluid that exhibits good correlation with serum levels of cortisol. One of the key advantages in using sweat as a detection modality for cortisol is the option to take advantage of emerging wearable technology. Sweat can be collected near-continuously with microfluidic systems, enabling *in situ* cortisol detection with wearable biosensors. However, the primary disadvantage of using a sweat based detection system lies in the difficulty of obtaining readings when a subject is not perspiring. Intensive exercise is often needed to stimulate sweat production, but the act of exercising triggers cortisol secretion, distorting measured values.^39^ Additionally, sweat based readings are not reliable over time due to residual cortisol from past perspiration, requiring complete removal of sweat from a wearable biosensor.

### Insights into Choice of Matrix/Sample

Overall, both salivary and sweat show a good correlation with free cortisol levels in serum,^40^ with the added advantage of being noninvasive. Additionally, salivary cortisol levels are synchronous with serum levels for up to 24 h with the important caveat that readings are skewed if the patient is on oral contraceptives.^41^ Thus, both fluids can offer a good detection modality for PoC/rapid diagnostic options for cortisol measurement. Interstitial fluid is another attractive sampling option, although it is more difficult to access compared to sweat or saliva. This can be circumvented by utilizing technologies such as microdialysis^38^ or microneedles.^42^ Interstitial fluid also has the unique advantage of being the only fluid that can be collected while a subject is asleep–both saliva and sweat fall short in this regard. The measurement of cortisol levels during sleep is a poorly understood yet critical parameter for a variety of tests such as Cushing’s and Addison’s replacement. Sleep cortisol measurements could also provide insights into psychiatric and inflammatory diseases.

## Overview of Established Technologies for Cortisol Detection

Throughout the history of cortisol detection, various methods have been deployed, each improving upon the disadvantages and limitations of the last. Currently, a wide range of assays are in use for clinical testing, including methods such as enzyme linked immunosorbent assays (ELISAs), radioimmunoassays (RIAs), fluoroimmunoassays, colorimetric analysis, bioluminescent probes, immunoassays, and lateral flow devices. The earliest reported measurements of cortisol involved fluorometric analysis experiments.^43^ However, the nonspecificity of these methods and their inability to distinguish between similar hormones (e.g., cortisol, corticosterone, and 11-deoxycortisol) made the technique redundant. These assays required expensive reagents, were time-consuming and required trained personnel.^44^

### Immunoassays

To overcome the limitations of specificity and sensitivity, radioimmunoassay’s were developed,^44^ with the promise of more convenient measurements and enhanced assay performance. These assays marked the beginning of antibody–antigen interactions for hormone detection, specifically, the use of monoclonal antibodies immobilized to a solid substrate. These early assays achieved limit of detection (LoD) of 8 ng/mL, representing a remarkable improvement compared to earlier fluorometric assays.^45^

Modern immunoassays, including ELISAs further improved upon radioimmunoassay methods by being easy to perform in the lab and by requiring small volumes of sample fluid. These assays typically combine antigen–antibody binding with fluorescent probes to estimate total cortisol concentrations, with the high specificity of antigen–antibody binding allowing accurate detection of small molecules. A wide range of cortisol immunoassays are available in the market, with most kits using a ELISA format for free cortisol detection. A competitive assay is used instead of the more common sandwich ELISA due to cortisol’s small size of 363 Da; cortisol has a single binding site, while a sandwich ELISA requires a minimum of two binding sites.

**Figure 2:**
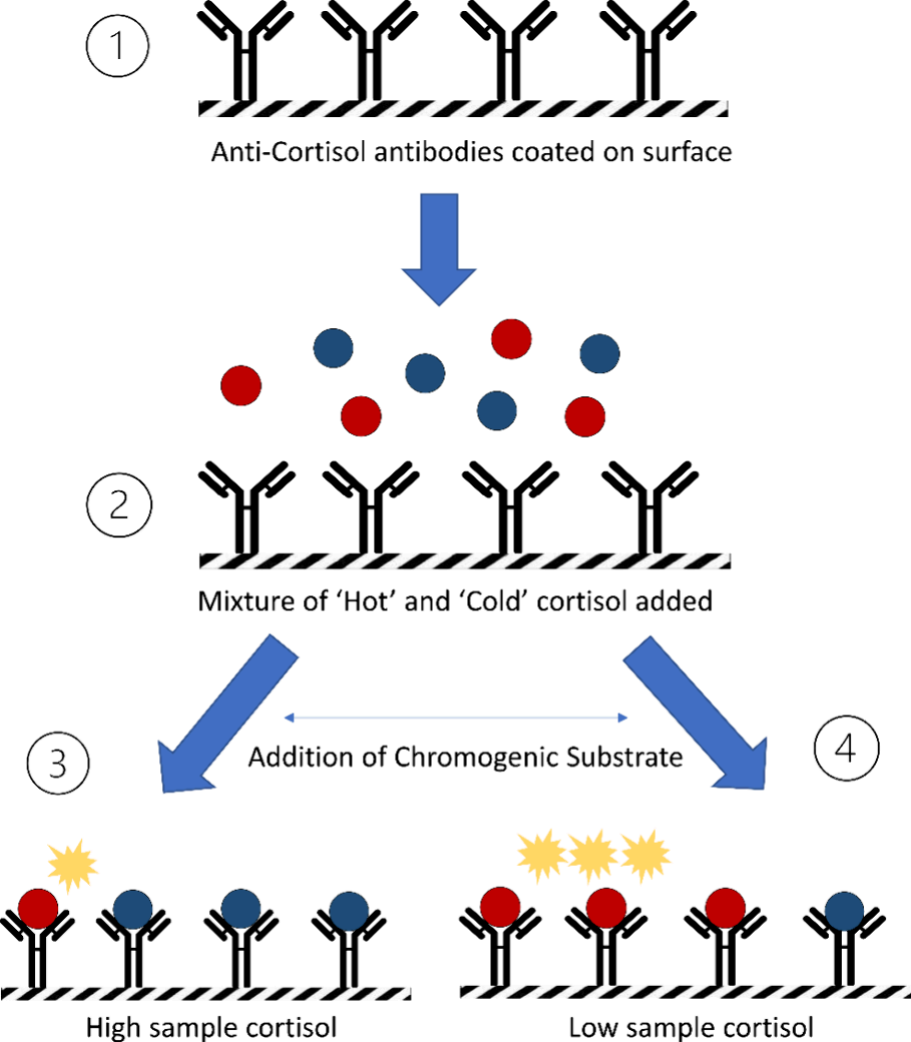
Schematic of competitive ELISA for cortisol quantitation. Anticortisol antibodies are coated onto a surface, such as a microwell. A mixture of sample (colored blue, containing unknown cortisol concentration) and a known quantity of labeled cortisol (colored red, conjugated to an enzyme, such as HRP) is added to the microwell. Upon addition of a chromogenic substrate, a drop in signal is observed if sample cortisol is greater than the quantity of labeled cortisol.

Figure 2 demonstrates the schematic for a competitive ELISA, in which a plastic surface such as a microtiter plate is coated with anticortisol antibodies. The test sample in which cortisol is to be measured (termed as “cold”) is added to the plate, along with a known concentration of cortisol conjugated to an enzyme (termed “hot”) such as horseradish peroxidase (HRP) or alkaline phosphatase (ALP). The two different cortisol species (labeled and unlabeled) compete for the same binding site of the immobilized antibody. Following an incubation period, a substrate is added which is oxidized by the enzyme, producing an amplified chromogenic signal. The intensity of the signal produced is inversely proportional to the amount of cortisol present in the sample, due to the “cold” cortisol occupying more binding sites than the “hot” cortisol, dampening the signal.^46^ While immunoassays for cortisol are widely used due to their relatively inexpensive nature and ease of use (compared to techniques such as mass spectroscopy), they can be hampered by issues of cross reactivity with other steroid hormones and are thus highly dependent on the quality of the monoclonal antibody.

### Mass Spectroscopy

The current gold standard for clinical cortisol measurement is mass spectroscopy (MS), a powerful technique routinely used for molecular characterization and the current best available tool to characterize free cortisol levels.^47^ MS is used as a highly specific method to analyze bodily fluids for cortisol, with liquid chromatography tandem mass spectroscopy (LC-MS/MS) able to distinguish between various steroid species and even synthetic steroids, allowing clinicians to observe steroid abuse.^48^ MS offers multiplexing allows simultaneous detection of multiple biomarkers of interest, a major advantage compared to immunoassays, in particular, microtiter-plate based assays. MS-based measurements are also unaffected by steroid cross-reactivity. However, while MS is precise and sensitive, the apparatus is bulky and the procedure is expensive due to column and solvent costs, relegating it to the purview of specialized laboratories and hospitals that can afford the systems and/or have trained personnel.^49^ These drawbacks make MS unattractive for use in PoC or rapid diagnostic applications.

### Shortcomings of Current Technologies

The rhythmicity of cortisol secretion is a defining characteristic for diagnostic testing that is difficult to capture with current sensing strategies. Due to the oscillating nature of serum cortisol levels, there is little diagnostic value obtained from a single point measurement; an observed value is only clinically relevant when measured in reference to the overall ultradian and circadian profile of cortisol levels throughout the day.

Currently, the diagnosis for hypercortisolism can be performed with a pharmacological suppression test or a midnight salivary test—when cortisol levels reach their nadir or lowest point. Complex conditions such as cyclic hypercortisolaemia need to be diagnosed with multiple consecutive midnight cortisol readings.^50^ Cortisol levels in such a condition can fluctuate between hyper and normal over a period of months—the number of tests required can place a large strain on both the individual and the healthcare system. The logistics of performing multiple, timed cortisol tests produces results that cannot be clinically interpreted with a high degree of confidence, in addition to the significant monetary and time cost spent on performing these tests. A rapid cortisol monitoring system could aid in the accurate diagnosis of adrenal insufficiency or hypercortisolism, while easing the burden on the healthcare system and the patient.^36^

The benefits of moving to a rapid diagnostic approach include frequent monitoring, turn-around time on the scale of minutes (as opposed to hours, as seen in clinical laboratories), less dependence on skilled technicians/devices and an overall reduction in cost of sensing per patient. Additionally, the technology associated with rapid/real-time sensing tends toward miniaturization, improving upon the portability and ease of use of the final device. This approach can also lead to multiplexing designs, where multiple streams of data are sampled simultaneously.^51^ Utilizing rapid diagnostics for hormones such as cortisol can be invaluable to both clinicians and patients to better understand hormonal biorhythms.

Additionally, a distinction between rapid and continuous sensing of cortisol should be made, particularly when measuring the hormones biorhythms. Rapid sensing can refer to multiple point measurements made over time, which can then be collated to produce a picture of overall hormone rhythm. Most PoC sensors would belong to this category of sensing, as rapid PoC testing is simpler to perform and can be carried out noninvasively.

The end-goal of precision medicine style diagnostics would be a continuous monitoring system, including devices such as continuous glucose monitoring (CGM) devices, where the device is in continuous contact with the analyte. However, in comparison to rapid sensing, continuous sensing is far more challenging to perform, due to a wide range of constraints. These can include, but are not limited to, saturation of the detection system, size of the device, biofouling, biocompatibility, and power source for the device. Each of these problems must be solved before a continuous cortisol biosensor can be developed and used in clinical practice. Some of the components of a cortisol biosensor that can be improved upon include the capture molecule, the fluid uptake system, and the sensing transducer.

## Capture Molecules for Cortisol Detection

### Monoclonal Antibodies

**Figure 3:**
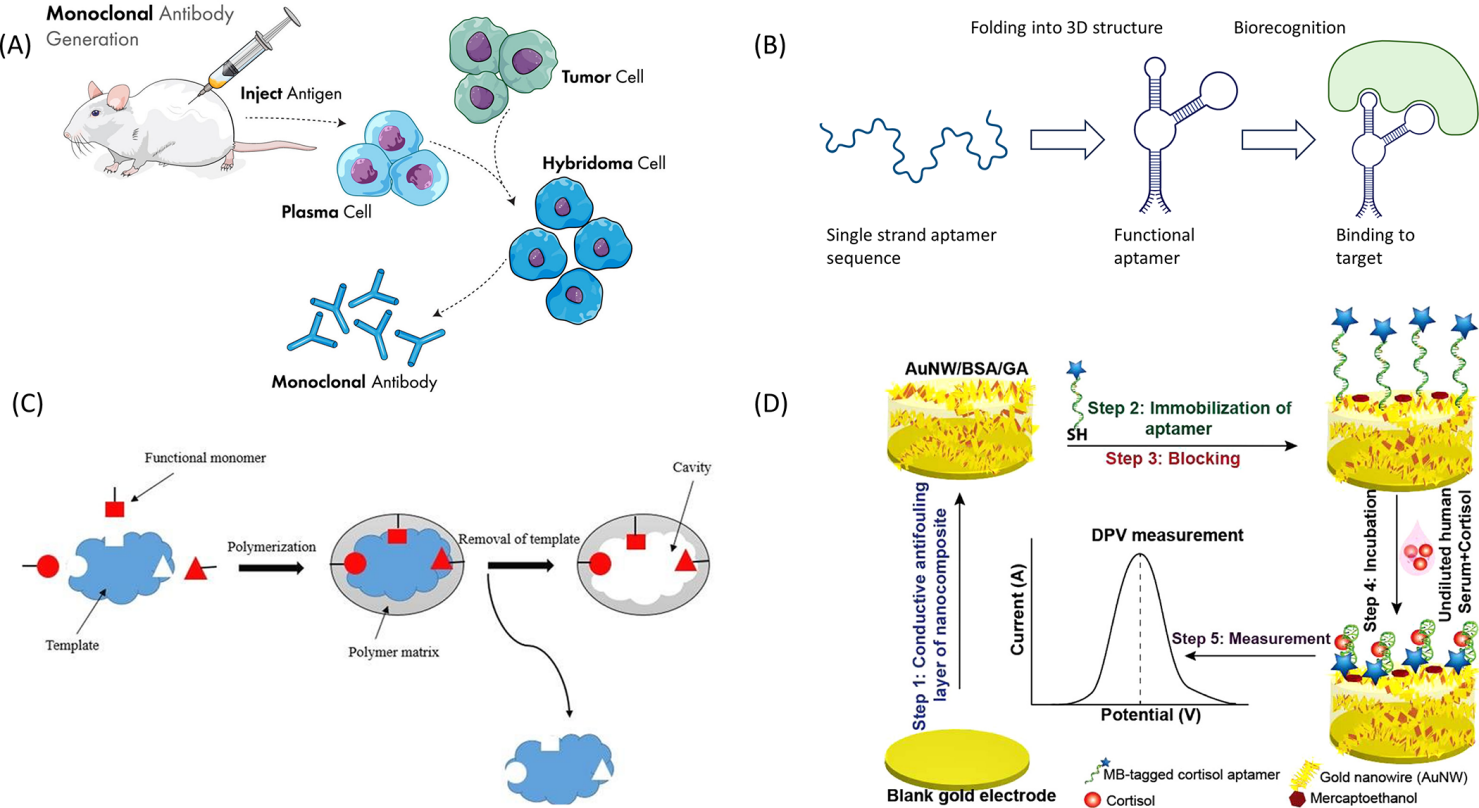
Various capture molecules used in cortisol assays. (A) Schematic of monoclonal antibody production via hybridoma technology. (B) Diagram of aptamer undergoing conformation change and binding to form an aptamer-target complex. Created with Biorender. (C) Schematic of MIP synthesis showing polymer molding and resultant cavity formation. Reproduced from ref (115) by Baker et al. Copyright 2015, *Iranian Biomedical Journal*. Licensed under CC BY 3.0 Deed|Attribution 3.0 Unported|Creative Commons. (D) Schematic overview of cortisol measurement using DPV, via conformation switching aptamers functionalized onto a gold nanowire substrate. Reproduced from ref (59). Copyright 2021, *ACS Omega*.

Monoclonal antibody-based immunoassays are ubiquitous due to their high specificity and affinity toward the target antigen.^52^ These antibodies can be reliably generated via hybridoma technology (schematic shown in Figure 3) to ensure consistency and reproducibility of assay results, with minimum batch variation. The affinity constant for a typical monoclonal antibody is 10^9^ L/mol which allows for nanomolar level detection of cortisol.^53^ It is also possible to create chimeric molecules with antibodies that act as “switches”.^54^ This molecular design made use of a fluorophore reporter pair, based on Förster resonance energy transfer (FRET), to measure changing cortisol concentrations from 1 nM to 100 nM.

Most cortisol diagnostic immunoassay kits make use of microtiter plates coated with monoclonal antibodies, as antibody clones have been extremely well characterized over the past decades and hybridoma technology has become well established. With trained personnel, an antibody-based assay for cortisol can be carried out in several hours and exhibit picogram level sensitivity for cortisol. The main disadvantages of using antibodies are the long generation time, expense, and in the case of cortisol, cross reactivity with other steroid hormones.^55^

### Aptamers

Aptamers are nucleotide-based capture molecules, used as an alternative to traditional antibody capture systems. Their advantage over antibodies includes a simpler method of synthesis, ease of chemical modification, high stability and overall cost effectiveness.^56^ Aptamers show excellent biorecognition ability, being able to selectively detect targets in complex fluids such as serum.^57^ This property is also useful in distinguishing cortisol from the many similar molecules that routinely interfere in antibody-based capture. Aptamers can be selectively oriented onto a desired transducer,^58^ making them ideal for coupling to electrochemical surfaces.

Additionally, aptamers can be configured to have conformation switching properties when they bind to cortisol, perturbing the charge transfer rate and enabling rapid, reagent free detection of cortisol. This is particularly viable when functionalized onto a conductive substrate such as gold, as shown in Figure 3.^59^ In that study, aptamers specific to cortisol were modified to be conformation-switching and were functionalized onto gold nanowires. The resulting biosensor demonstrated a LoD of 0.2 pg/mL while sampling from serum, a sample fluid with significant matrix interference.

### Molecularly Imprinted Polymers (MIPs)

Traditional capture molecules used for cortisol detection are monoclonal antibodies and aptamers. In recent years, molecularly imprinted polymers (MIPs) have emerged as an inexpensive and versatile tool for cortisol capture,^60−62^ retaining high specificity while being cheaper to synthesize and functionalize. Figure 3 shows the process of MIP synthesis via the process of molecular imprinting, wherein a template molecule is incorporated during the polymerization of a chosen monomer. After polymerization, the template molecule is removed, leaving behind cavities in the polymer matrix that are complementary in shape, size, and functional groups to the target molecule. These cavities act as selective recognition sites for the target molecule.

The recognition sites allow high affinity binding to the analyte, avoiding the common issue of cross-reactivity with similar targets. By selecting the appropriate monomeric unit of the polymer, recognition sites can be constructed, leading MIPs to be called “artificial receptors”.^63^ Due to their high customizability and potential for functionalization, MIPs can be easily coupled to electrochemical sensing systems in a wearable form.^64^ This type of sensor achieved rapid (<2 min) detection of cortisol with an LoD of 8 ng/mL, typical of cortisol ranges in sweat.^65^

MIPs have also been used in fluorescent competitive binding assays, demonstrating a very low LoD of 0.001 pg/mL.^66^ The cross-linked MIPs used in this study had a *K*_a_ value of 2.3 × 10^11^ M^–1^, 2 orders of magnitude higher than *K*_a_ values for anticortisol monoclonal antibodies.

## Emerging Biosensing Platforms Relevant for Cortisol Measurement

In recent years, multiple biosensing technologies have emerged, to address the limitations of current cortisol detection modalities and to improve upon current sensing strategies. Next generation cortisol sensing platforms incorporate multiple technologies under a unified umbrella package to provide accurate, sensitive, and rapid small analyte detection. Overall, two main biosensing modalities are emerging, one being miniaturized microfluidic devices and the other electrochemical sensing. Often, these two are combined to explore the best of two worlds: label-free detection of electrochemical sensing with miniaturized,^67^ power-free fluid flow of microcapillary microfluidic devices.^68^ These will naturally become increasingly more relevant as progress is made in terms of miniaturization and automation of the device while improving clinical performance such as LoD.

Next generation biosensing modalities should be readily adaptable to healthcare models of the future—decentralized, personalized, and aligned with precision medicine. This places further demands on certain characteristics of the sensor, summarized in Table 2. Adaptability of the technology to rapid or continuous formats, the affordability per test and potential to scale the test are all important properties that should be considered. Another aspect to consider is the “integration potential” of a particular modality. This refers to how easily a particular technology can be integrated with other strategies, to offset the disadvantages of one or the other. An example of this could be using immunoassays with microfluidic systems, via surface functionalization chemistry. This could enable detection of cortisol using low volumes and rapid incubation times, while maintaining the sensitivity and selectivity of immunoassays.

**Table 2:** Compared Characteristics of Various Next Generation Biosensing Strategies

Biosensing strategy	Adaptable to PoC format	Adaptable to continuous format	Affordability per test	Scalability	Integration potential
Immunoassay	√				√
Electrochemical sensing	√	√	√		
Spr	√				
Wearable devices		√		√	√

### Microfluidics

Microfluidics refers to the utilization of small volumes of sample in micron scale wells/channels, facilitating an easier control of physical and chemical processes.^69^ With optimization, microfluidic devices have demonstrated orders of magnitude increases in mass-transfer over traditional laboratory-scale reactors.^70^ This is particularly critical when attempting to design rapid diagnostics; fast mixing of the reagents and short reaction times are key. This is due to the enhanced mass transfer obtained from high surface to volume ratio, short diffusion distances, good temperature control and standardizable flow patterns. The low sample volumes observed in most microfluidic biosensors helps to minimize the need for human intervention during the assay analysis and avoids the complications involved with bulk processing of liquid.^71^

In addition to incorporating multiplexing and miniaturization, certain models of microfluidic devices can also be created cheaply with highly scalable production methods such as melt extrusion.^72^ Simple flow through tubes can be coupled with printed electrodes and functionalized with anticortisol antibodies to create a low-cost PoC device.^61^ The precision and speed offered by these devices have the potential to create next generation sensing platforms for small molecule detection, in a sensitive yet affordable package. Indeed, droplet-based microfluidic devices have been coupled with magnetic beads to create an electromagnetic “bead-based” immunoassay for cortisol detection,^73^ performing near real-time measurements of the hormone in a rapid fashion, while only requiring a very small quantity of sample fluid (350 nL). It is worth noting that many wearable devices for cortisol detection employ microfluidic channels and structures for sample uptake, highlighting their versatility, and ease of use.

**Figure 4:**
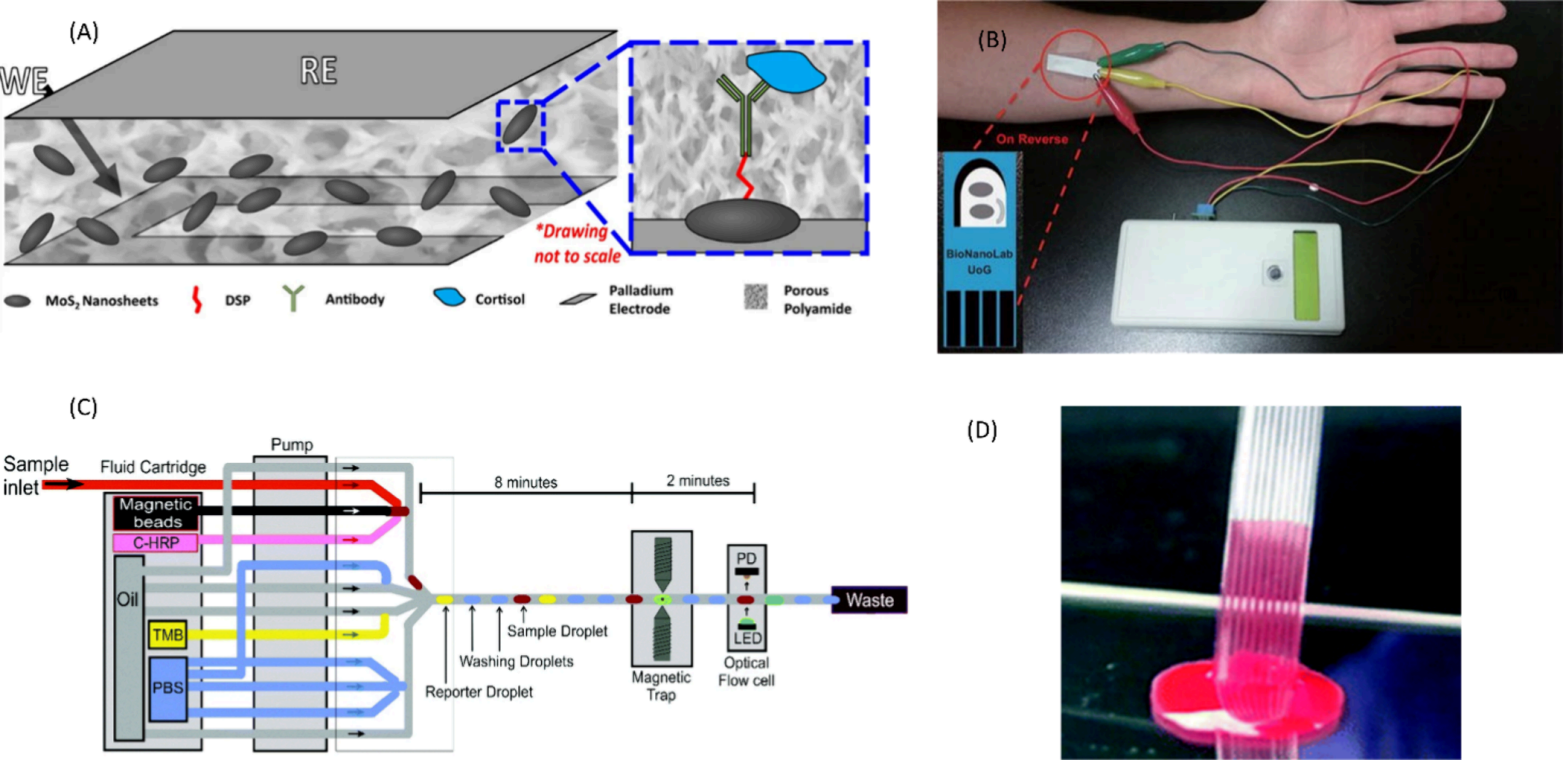
(A) MoS_2_ based electrochemical sensor for cortisol. A polyamide membrane contains the working and reference electrode, nanosheets containing conjugated antibody are embedded within. Reproduced from ref (76) by Kinnamon et al. Copyright 2017, *Scientific Reports*. Licensed under CC BY 4.0 Deed|Attribution 4.0 International|Creative Commons. (B) Prototype electrode for noninvasive sampling of cortisol, shown attached to portable hand-held potentiostat and associated Bluetooth connection. Reproduced from ref (75) by Tuteja et al. Copyright 2018, *Nano-Micro Letters*. Licensed under CC BY 4.0 Deed|Attribution 4.0 International|Creative Commons. (C) Microfluidics used for the assembly of a “droplet-train”, bead-based immunoassay for cortisol. Reproduced from ref (116) by Evans et al. Copyright 2021, *Analyst*. Licensed under CC BY-NC 3.0 Deed|Attribution-NonCommercial 3.0 Unported|Creative Commons. (D) Multi channel microcapillary film used to create a “lab on a stick”. Reproduced from ref (66) by Reis et al. Copyright 2016, *Royal Society of Chemistry*. Licensed under CC BY 3.0 Deed|Attribution 3.0 Unported|Creative Commons.

Figure 4 demonstrates the bead-based immunoassay configuration as well as transparent fluoropolymer microfluidic strips used for optical biosensing. An exciting prospect for cortisol detection is the use of multibore microcapillary film (MCF), which shows excellent optical properties while being high throughput.^72^ The advantages of microfluidic based reactors are highlighted in such a configuration, which could be easily adapted for cortisol sensing.

### Electrochemical Sensing

Electrochemical sensing of biological samples is an area of high interest, with techniques like cyclic voltammetry (CV), differential pulse voltammetry (DPV), and electrochemical impedance spectroscopy (EIS) routinely used to assess parameters such as cell growth^74^ or binding affinity. With more research groups now having access to micro/nano fabrication facilities, there has been a rapid increase in the type and number of electrochemical devices created, particularly in the field of wearable health trackers.

An advantage of using electrochemical sensing is the ability to construct label-free assays, using methods such as chronoamperometry or EIS. A chronoamperometric detection method was used in a graphene embedded screen-printed electrode to produce a biosensor for cortisol detection, with a LoD of 0.1 ng/mL.^75^ The assay took less than a minute to complete and utilizes a portable potentiostat to enable point-of-care (PoC) detection. In another study, molybdenum disulfide (MoS_2_) nanosheets coated with anticortisol monoclonal antibodies were used to determine cortisol concentrations from sweat with a LoD of 1 ng/mL, using EIS.^76^ Other label-free sensors use gold microelectrodes were functionalized with dithiobis(succinimidyl propionate) to produce a cortisol sensor based on EIS, with an extremely low LoD of 0.0004 pg/mL.^77^

Additionally, by coupling electrochemistry with immunoassays, high-fidelity PoC devices can be created. These devices can aid the transition clinical/laboratory hormone measurements to a precision medicine approach, with individuals able to buy off the shelf devices for personal use. For example, graphene oxide-based electrodes have been used in conjunction with an immunoassay capture system to measure cortisol in a label-free manner, using a hand-held potentiostat for easy measurement of the device readings.^75^

## Rapid Cortisol Technologies

### Lateral Flow Devices

**Figure 5:**
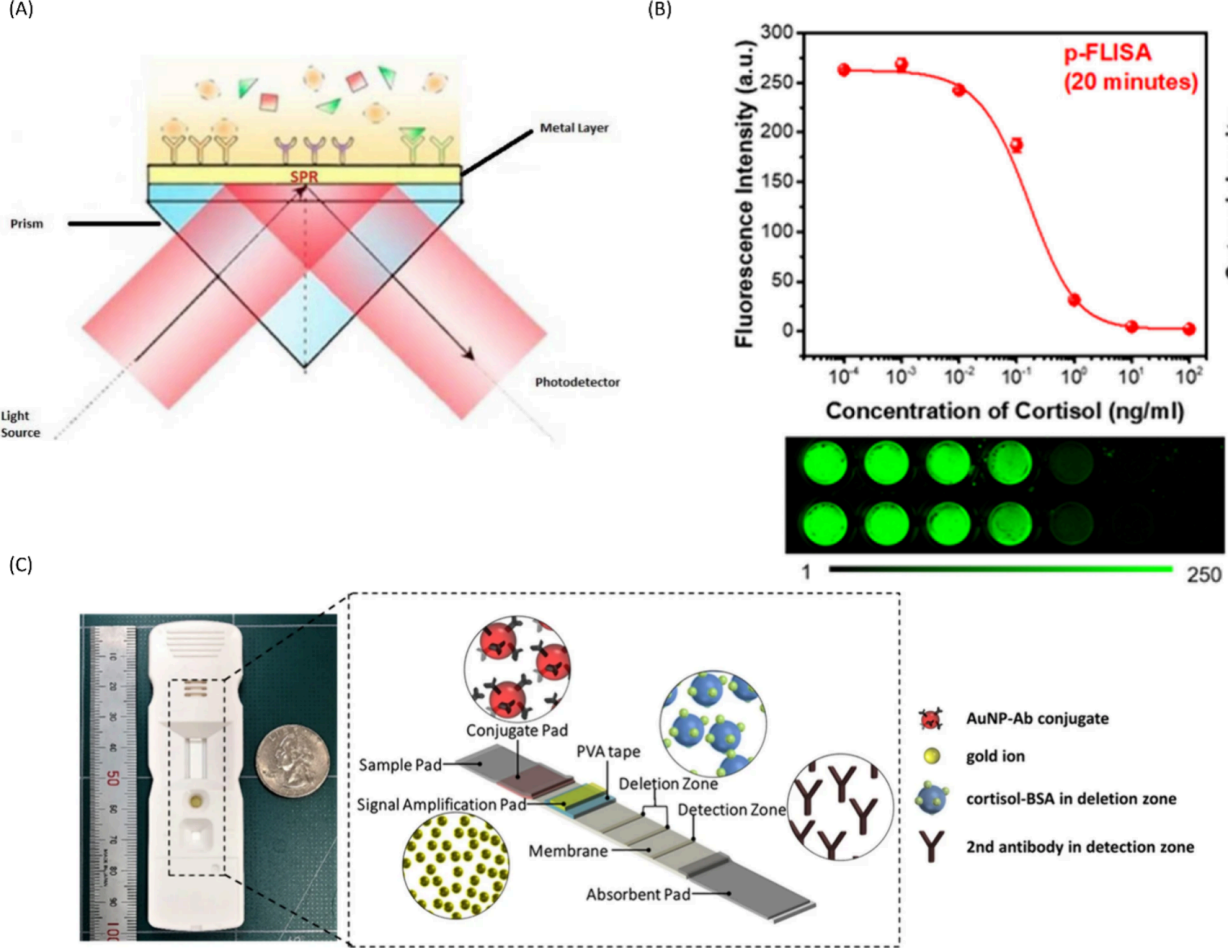
(A) Schematic representation of SPR. Reproduced from ref (117) by Deng et al. Copyright 2023, *Sensors and Actuators B: Chemical*. Licensed under CC BY 4.0 Deed|Attribution 4.0 International|Creative Commons. (B) Schematic of a cortisol LFIA, showing construction of the sensor with various immunoassay components. Reproduced from ref (81) by Oh, HK et al. Copyright 2021, *Scientific Reports*. Licensed under CC BY 4.0 Deed|Attribution 4.0 International|Creative Commons. (C) Dose dependent curse in PEC-QD system showing decrease in fluorescence as cortisol concentration increases. Reproduced with permission from ref (89). Copyright 2022, *Biosensors and Bioelectronics*.

Lateral flow immunoassays (LFIAs) exemplify the ideal PoC device—easy to handle, low cost, and power free, being driven by capillary action,^78^ which aligns well with ASSURED criteria used for development of PoC testing. While in general they inherently suffer from constrained LoD and difficulty in quantitation, this can be remedied by using signal amplification methods such as coupling the analyte to fluorescent nanoparticles or using enzyme-based colored reactions. Figure 5 shows a typical LFIA structure, demonstrating the miniaturizable nature of the device. Having played a key role in diagnosis through the COVID-19 pandemic, LFIAs have also been used in small molecule detection, such as cortisol. Simple, competitive-chemiluminescent LFIAs have been designed for cortisol detection, with a LoD of 0.3 ng/mL, while being able to be read by a smartphone camera.^79^ Indeed, the ability to couple smartphones to LFIAs as a portable and ubiquitous “reader” is serendipitous, with reference cards having been developed for capture normalization.^80^ Additionally, more advanced variants have also been developed such as a trap LFIA,^81^ which enabled the quantification of salivary cortisol with a LoD of 9.1pg/mL.

### Surface Plasmon Resonance

Surface Plasmon Resonance (SPR) is a relatively new tool for biosensor development that enables the detection of molecular binding events such as antigen–antibody binding. Figure 5 demonstrates the principle of SPR. Initially, the light beam is directed toward the SPR coupler to stimulate the oscillation of electrons on the metal surface (surface plasmons). Interactions of light with these surface plasmons result in a dip in the intensity of reflected light.^82^ Subsequently, the detector captures the diffracted or reflected light. A small change in refractive index at the interface of the metal-dielectric induces a corresponding modification in the effective index of the surface plasmon. Consequently, any variations in the refractive index can be detected through changes in the intensity, angular, or wavelength of the emitted spectrum of the surface plasmon.

This technique has been used in the development of several indirect ELISA immunoassay for cortisol, where anticortisol antibodies interacted with a surface modified with a cortisol analogue and the binding events were then measured via SPR.^83,84^ By making use of a BIAcore SPR system with microfluidic cartridges, the ELISA incubation time was lowered from 2 h to 25 min, while maintaining a LoD of 0.038 pg/mL.

The use of SPR for cortisol detection has also been tested with plastic optical fibers coated in gold/palladium (Au/Pd). Anticortisol antibodies were bioconjugated to the Au/Pd using EDC-NHS coupling and upon addition of cortisol, a red shift was induced in the SPR signature. The sensor demonstrated a remarkably LoD of 1pg/mL, along with the possibility of using inexpensive plastic optical fiber cables as a chassis for a scalable biosensing device.^85^

SPR offers a great deal of flexibility in the design and functionalization of a biosensing surface, with gold, zinc, palladium, and even coated plastics being explored as possible substrates.^86^ These metal surfaces can be engineered in unique ways, such as a nanosensing cuvette that only requires half an hour for a reading as opposed to multiple hours as per a traditional immunoassay.^87^ SPR can also facilitate the label free detection of cortisol while discerning between other hormones by using relative shifts in the refractive index, while maintaining a LoD of 0.1 nm.^88^ While current apparatus for SPR is not feasible for home testing, it could find a presence in clinics and hospitals, enabling rapid detection of cortisol in a clinical setting.

### Advanced Immunoassays and Supramolecular Chemistry

The main challenges associated with traditional immunoassays are in general, weak signal, constrained LoD, possibility of matrix interference and cross reactivity. Acknowledging these challenges, next generation immunoassays are under development, taking advantage of technologies, such as quantum dots (QD), photoelectrochemical sensing (PEC), electrochemistry, and fluorophore-based emission. Wang et al.,^89^ integrated an ultrabright label, plasmonic-fluor, with anticortisol monoclonal antibodies, to develop a rapid, highly sensitive cortisol assay, with a LoD of 2 pg/mL, enabling quantification of cortisol levels within 20 min. Additionally, the assay was multiplexed, being able to detect cortisol and fluorescein simultaneously.

Figure 5 shows data from an advanced immunoassay constructed with QDs and PEC sensing. PEC sensing employs the use of multiple chemical reaction steps to amplify the signal obtained from antigen–antibody binding, utilizing reactions such as enzyme-mediated catalysis, chemical redox cycling, nanomaterial mediated electron excitation, to name a few. As a step up from electrochemical bioanalysis, PEC possesses fast response times, good signal-to-noise ratio and high sensitivity.^90^ A competitive assay based on PEC immunosensing was developed with an extremely low LoD of 0.06 pg/mL.^91^ This type of “signal-on” immunoassay could become routine as PEC based reactions become more commonplace. As supramolecular techniques grow more refined, with further understanding of coupling reactions, sensitive and discerning assays for cortisol can be developed for rapid cortisol detection.

### Quantum Dots

Quantum dots (QD) are nanoscale structures exhibiting strong photoluminescence quenching effects, while being easily modifiable with conjugate groups/capture molecules such as antibodies or aptamers. By conjugating QDs with anticortisol aptamers and binding the complex to magnetic nanoparticle labels, a QD cortisol sensor^92^ was developed, showing LoD of 1 nM (aptamer based) and 100 pM (antibody based). QD based sensing can also be carried out with nonlabeled versions utilizing microwave treatment, with a LoD of 16 ng/mL.^93^ Additionally, QDs can be combined with PECs to achieve extremely low LoDs. However, as QDs are a young technology, cost, and stability of the reagents are key challenges to be overcome.

## Continuous Cortisol Technologies

### Wearables

Wearable electronics are ubiquitous in modern society, from smart watches to continuous glucose monitors. They track a wide range of parameters, are rugged in design and operation, and are becoming more economically accessible as their development progresses. One of the biggest expected advantages of ubiquitous wearable technology is the ability to empower patients to take charge of their own health, while reducing the burden of testing and diagnostics on centralized healthcare systems.^94^ This is an aspect that will certainly polarize healthcare practitioners, but one that CGM has created an inspiring precedent for. The development of wearables for cortisol detection is an extremely popular field, with many different modalities being explored.^95−97^ Most wearables use immunoassays coupled to an electrode, i.e., antibodies are conjugated onto an appropriate surface and their subsequent binding to cortisol generates a charge transfer event, which can then be measured.^98^ As wearable devices for saliva are difficult to attach onto an appropriate surface, sweat is often used as the sampling fluid, with the device attached to the forearm/upper arm.^97^

**Figure 6:**
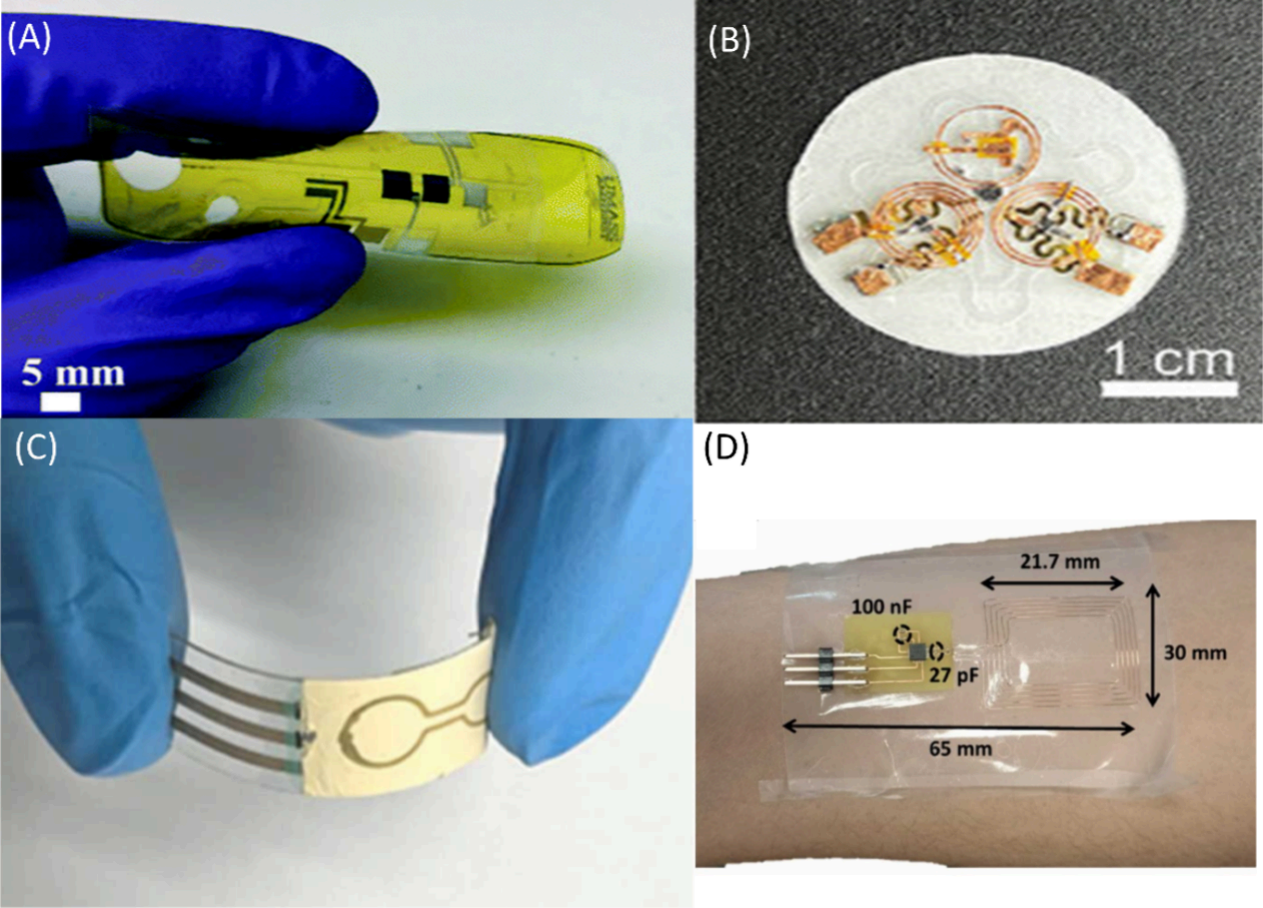
Various wearable electrochemical sensors for the continuous measurement of cortisol. (A) “Band-aid” form factor electrode on a printed graphene electrode, utilizing antigen–antibody binding. Reproduced with permission from ref (61). Copyright 2021, Royal Society of Chemistry. (B) A multiplexed sensor patch capable of sensing cortisol, temperature and pH from sweat, making use of aptamer-cortisol binding. Reproduced from ref (100) by Dong et al. Copyright 2022, *Advanced Functional Materials*. Licensed under CC BY 4.0 Deed|Attribution 4.0 International|Creative Commons. (C) Carbon nanofiber-based sensor with molecular imprinted polymer (MIP) as cortisol capture molecule from sweat. Reproduced with permission from ref (118). Copyright 2023, *Sensors and Actuators B: Chemical*. (D) A paper based magnetic bead cortisol immunosensor using sweat as a sample fluid. Reproduced with permission from ref (99). Copyright 2023, *Sensors and Actuators B: Chemical*.

Figure 6 summarizes some of the different approaches used in the designing of wearable sensors differing in redox chemistry, sample collection, measurement, electrode substrate, and form factor. Other approaches include a “band aid” form-factor electrode with a microfluidic sample delivery system, utilizing gold nanoparticles conjugated to anticortisol antibodies, packed onto a printed graphene electrode using a linker molecule.^61^ Another innovative device includes the use of filter paper in the form of microfluidic channels for sample delivery onto screen printed electrodes, for use in sweat cortisol measurement during physical exercise.^99^ Also using sweat, a battery free, skin-adhesive wearable was designed, making use of a “two-part” resonance circuit model to sense cortisol with an LoD of 0.1 ng/mL.^100^

One of the biggest challenges in using wearable technology for sweat based cortisol analysis is ensuring the continuous supply of sample fluid. Rate of human sweat formation is low in nonexercising individuals and even microfluidic devices would find it difficult to carry enough sweat to the cortisol sensor. This difficulty can be circumvented to some degree by using reverse iontophoresis, a method which applies a localized microcurrent to induce sweat formation.^101^ However, the challenge of residual cortisol remaining from evaporated “old” sweat remains to be solved. Thus, a sweat-based cortisol wearable must be powered to induce iontophoresis, incorporate a wicking system and be miniaturizable—to not be a hindrance to the user.

### Microdialysis

In microdialysis technology, a semipermeable hollow probe of appropriate pore size (normally ranging from 20–150 kDa) is inserted subcutaneously via a catheter, allowing continuous sampling of interstitial fluid. The probe is perfused with a saline solution at a low fluid flow rate, to minimize the impact of the operation upon the extracellular space. By selecting an appropriate membrane size (20 kDa), larger analytes such as cytokines can be eliminated from the sample space.^102^ Microdialysis is minimally invasive compared to techniques, such as venepuncture, with tissue damage due to probe insertion is virtually nonexistent.^103^ Additionally, It has been shown that free cortisol levels can be measured in interstitial fluid, making it a useful technique when considering continuous measurement of cortisol.^104,105^

**Figure 7:**
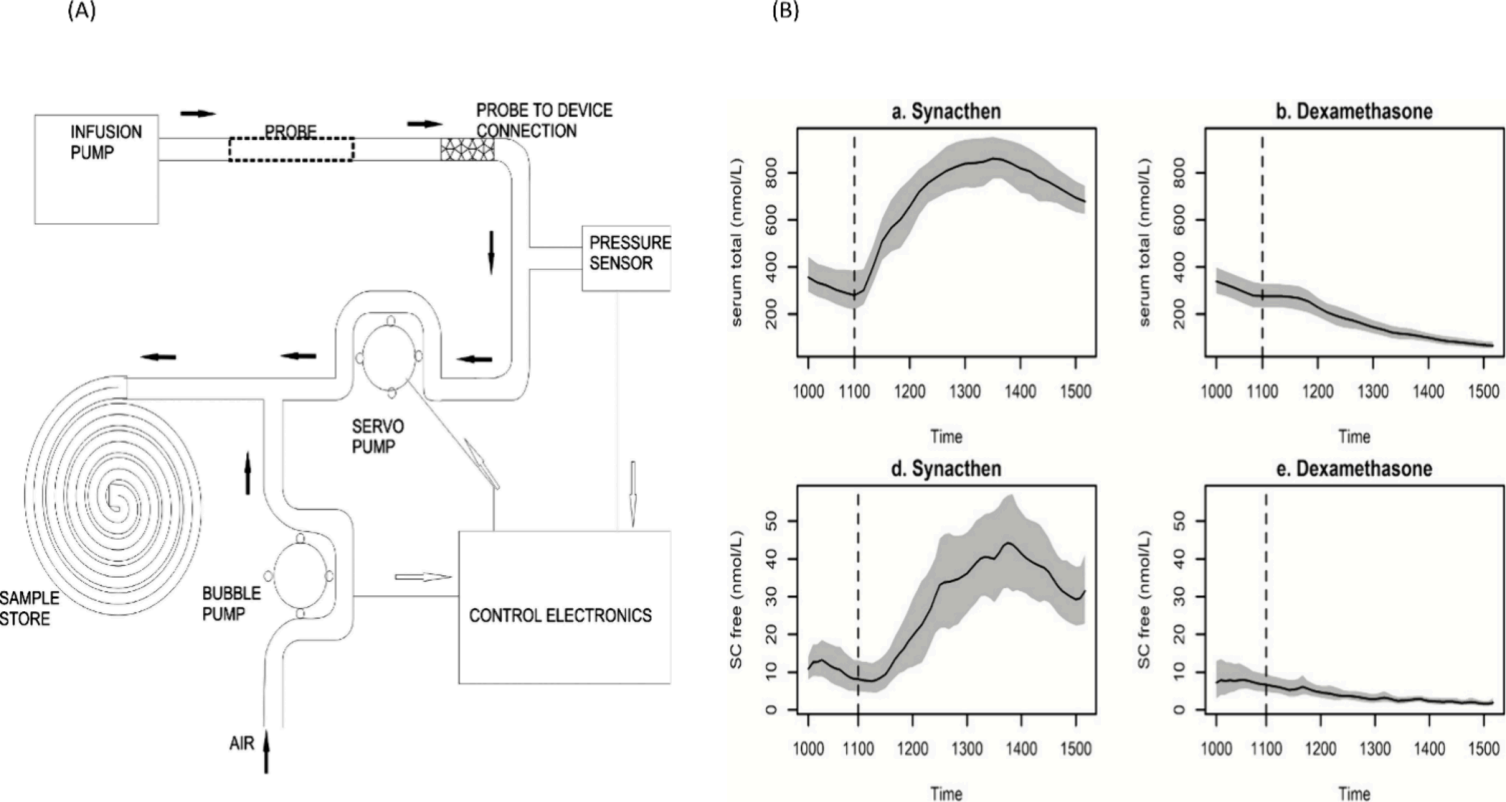
(A) Microdialysis pump system schematic depicting sample collection and storage. Reproduced with permission from ref (106). Copyright 2013, *Journal of Medical Engineering and Technology*. (B) Cortisol levels obtained from operation of the previously described pump. Panels a and d depict cortisol level spikes in response to treatment with the compound Synacthen, whereas panels b and e show depressed cortisol levels due to treatment with dexamethasone. Reproduced with permission from ref (107). Copyright 2019, *The Journal of Clinical Endocrinology & Metabolism*.

Recently, an automated microdialysis system for 24 h measurement of cortisol was developed, shown in Figure 7.^106^ By utilizing a double pump setup and microcontrollers, the system was able to collect samples over the full course of 24 h, which were then analyzed via immunoassay. A key advantage of the microdialysis system is the easy access to interstitial fluid, an analyte better suited for continuous sensing than saliva or sweat. Additionally, dynamic changes in cortisol levels were monitored, in response to various compounds that spiked or lowered cortisol secretion rates.^107^ The ability to monitor fluctuating cortisol levels in real-time is a paradigm shift from traditional measurement modalities. The coupling of microdialysis to immunoassays can pave the way for the next generation of continuous cortisol sensing. The ability of microdialysis based sensors to remain in constant contact with the analyte fluid enables the construction of reversible sensors.^38^

The current limitation of microdialysis devices is that they are only capable of sample collection; processing the collected samples still requires the use of immunoassays or LC-MS. Coupling a “quantitation module” onto a microdialysis system could render the setup too bulky to carry, essentially relegating the system to clinic/hospital setting. A wearable microdialysis system that can quantify collected samples “on the fly” would carry over the advantages of wearable sweat-based cortisol sensors into interstitial fluid sampling, an exciting avenue of research.

## The Future of Cortisol Sensing

With the escalating prevalence of stress-associated endocrine diseases and disorders in contemporary society, there is an imminent need for rapid and/or dynamic quantification of cortisol levels. However, significant changes to current cortisol measurement modalities are needed. Key challenges that are currently not addressed by traditional cortisol sensing include the time taken per test, the rhythmicity of cortisol secretion and the lack of PoC testing options. Additionally, standards for cortisol measurement differ greatly between individuals, as well as between age and population subgroups, with children having subtly different secretion rhythms to adults. Standardization of cortisol levels in different fluids is difficult due to the unique biorhythm of an individual, which can lead to difficulties when attempting to diagnose a complex condition, such as Cushing’s, using only cortisol values. These limitations present many opportunities for the development of novel cortisol detection modalities.

A diverse range of cutting-edge biosensing platforms and technologies herein reviewed, including microfluidics, wearable electronics, electrochemistry, advanced immunoassays, molecularly imprinted polymers (MIPs), and microdialysis, are under investigation for cortisol monitoring. These innovative approaches offer the potential for improved LoD through improved biorecognition elements, optimized analyte fluid uptake, and reduced sample volume requirements.

With the overall trend of cortisol detection moving toward rapid or continuous monitoring, it seems likely that a wide array of PoC devices will become readily available for public use, in the form of quantitative LFAs or microfluidic based dipsticks using saliva or sweat samples. This bypasses the invasive nature of serum-based testing, while offering timely and accurate readouts without the requirement of pretreatment steps or external power. This would be transformative in allowing clinicians to monitor cortisol levels in patients being treated with glucocorticoids or allowing patients with Addison’s to monitor their cortisol levels. Rapid cortisol tests also benefit from unrestricted use of sample fluid as an analyte, as sustained contact with the sample is not required. Indeed, it might even be possible to manufacture cortisol tests at a similar scale to COVID-19 testing solutions, while still retaining quantitation capability.

However, it may prove difficult to understand cortisol’s rhythmicity through rapid testing alone. With a strong link between sampling, nature of the matrix, and LoD of the test, the burden of performing multiple tests through the day could quickly become tedious and cost inefficient- even without considering the necessity for measurements made during sleep, when measurement of cortisol becomes clinically most relevant. Additionally, cortisol assays suffer from cross-reactivity to other steroid hormones such as prednisolone, a commonly prescribed corticosteroid medication. In addition to high selectivity, the assay would also need to be highly sensitive, due to the low concentrations of cortisol in sweat and saliva. Capture molecules such as MIPs could prove extremely useful and outperform their antibody counterparts, due to the intensely selective nature of their formation.

While continuous monitoring would deliver an informative, real-time picture of hormonal biorhythm, such sensors are more complex in their design and development. New challenges, such as powering the device, sensor fouling, matrix interference effects and longevity of the device, must be addressed. In particular, fouling of sensor services is a critical concern in the developmental pipeline of continuous biosensors—constant contact with the complex matrices of biological fluids performance and greatly reduces sensor lifespan. The importance of choosing an appropriate matrix becomes clear, further driven by the difficulty in utilizing saliva or sweat for continuous sensing. The importance of choosing an appropriate matrix becomes clear, as the utilization of fluids such as saliva and sweat are difficult to adopt to a continuous sensing format. Each technology platform brings its own unique advantages and drawbacks, e.g., utilizing a microdialysis-based approach enables access to interstitial fluid but would require the patient to wear a catheter and microdialysis pouch for over 24 h. Using microfluidics for fluid uptake lowers the volume of sample required but necessitates careful modification of the polymer tubing substrate to avoid hormone smearing during sample collection. These challenges can be mitigated by exploring the synergies between various technologies; for example, integrating microdialysate flowthrough into an impedance-based chip via multichannel microfluidics systems would allow for multiplexed, real-time tracking of cortisol levels. Improvements in material science, both in the synthesis and modification of biosensor substrates and capture molecules, will play a key role in building more robust sensors that can perform well under *in vivo* conditions. Rather than focusing only on lowering the LoD, often in synthetic biofluid samples, more importance should be given to establishing reproducibility and linear dynamic ranges in biological matrices. Ultimately, the type of sensing strategy employed should be synergistic with the intended format of measurement.

The scalability of testing modalities is also an important factor to be considered, especially in the context of public health. The COVID-19 pandemic brought this issue to the fore, where cheap, scalable, PoC LFAs became widely accessible and drew public attention to PoC testing. Next generation biosensing strategies need to be equally accessible to the public, such as in the case of continuous glucose monitors, to be utilized to their full potential and enable true quantitation of hormone levels.

A wide variety of biosensor designs and proof-of-concept devices have been proposed for rapid cortisol quantitation. However, user-friendly biosensors offering capabilities for rapid or continuous cortisol quantitation or monitoring are yet to be fully realized. By maintaining a clinician and consumer-focused perspective, the utilization of rapid and continuous cortisol biosensors can positively impact stress management. As biosensing materials and conjugate chemistry continues to mature, real-time continuous monitoring of cortisol, alongside other hormones, could become routine in healthcare, empowering patients to take charge of their own health and providing clinicians with powerful tools for fundamental research.

Beyond cortisol, such devices could unlock new avenues for measurement of other hormones such as circulating testosterone, estrogen, thyroid hormones, or other hormones in the HPA axis (such as ACTH or CRH) and could be immensely valuable from both a clinical and user perspective. A valuable outcome for hormone testing would be multiplexed sensors that can simultaneously quantify several hormones in the same device/test strip, leading to extremely high throughput diagnostics. The benefits of having a well-informed population could go a long way toward reducing the diagnostic burden on primary healthcare organizations, while ushering in an era of personalized and precision medicine.
